# The complete mitochondrial genome of *Appias albina* (Lepidoptera: Pieridae) and phylogenetic analysis

**DOI:** 10.1080/23802359.2020.1721347

**Published:** 2020-02-03

**Authors:** Xiao-Dong Li, Wei Zhang, Wan-Tao Rong, Yi-Feng Wang, Ran Li

**Affiliations:** a School of Chemistry and Bioengineering, Hechi University, Yizhou, Guangxi, P. R. China;; b The Key Laboratory of Jiangsu Biodiversity and Biotechnology, College of Life Sciences, Nanjing Normal University, Nanjing, Jiangsu, P. R. China

**Keywords:** Lepidoptera, *Appias albina*, mitogenome, phylogenetic analysis

## Abstract

The complete mitochondrial genome of *Appias albina* (Lepidoptera: Pieridae) was firstly sequenced and characterized in our study. The total length of mitogenome is 15,193 bp and contains 13 protein-coding genes, 2 ribosomal RNA genes, 22 transfer RNA genes, and 1 A + T-rich region. The overall nucleotide composition was 38.4% of A, 8.0% of G, 40.9% of T, and 12.4% of C. Phylogenetic tree was reconstructed using Bayesian Inference (BI) to validate the taxonomic status of *A*. *albina*, exhibiting the close relationship with *Appias remedios*.

The species of genus *Appias* belongs to the subfamily Pierinae, within the family Pieridae of the order Lepidoptera (Braby et al. 2010). At present, only the complete mitochondrial genome of *Appias remedios* was reported about this genus (Zhang et al. 2019). In this article, we determined and described the mitogenome of *Appias albina* in order to obtain basic genetic information about this species.

The specimens of *A. albina* were collected from Huanjiang, Guangxi province, China. All samples were stored in 95% ethanol at temperature of –20 °C and deposited in the Museum of Insects of Hechi University (the voucher No. L386), Yizhou, Guangxi. Whole genomic DNA was extracted from abdomen of each specimen using a Wizard^®^ Genomic DNA Purification Kit (Promega, Madison, USA) according to the manufacturer’s instructions. The mitogenome of *A. remedios* (GenBank accession No. MF576060) was employed as the reference sequence (Zhang et al. 2019). Certain pairs of universal primers for butterfly mitochondrial genomes were used for polymerase chain reaction (PCR) amplification (Simon et al. 2006). Then, PCR products were sequenced using primer-walking strategy from both strands. Mitochondrial genome was assembled by SeqMan program from DNASTAR (Burland 2000). The positions of RNA genes were predicted by the MITOS (Bernt et al. 2013) and the locations of protein-coding genes were identified by comparing with the homologous genes of other closely related species.

The complete mitogenome of *A. albina* (Genbank accession No. MN643595) was sequenced to be 15,193 bp in size. The mitogenome consisted of 13 typical protein-coding genes, 22 transfer RNA genes, 2 ribosomal RNA genes, and 1 A + T-rich region, which is similar to the typical mitogenome of other insects (Cameron 2014). Like other pierid mitogenomes, 24 genes are encoded on the H-strand and the other 13 lie on the L-strand. The overall nucleotide composition was 38.4% of A, 8.0% of G, 40.9% of T, and 12.4% of C. All PCGs, except COI, initiated by typical ATN codons. Two PCGs were ended with a single T right ahead of tRNA genes and the other 11 genes harbored the complete termination codon TAN.

In order to validate the new determined sequence, whole mitochondrial genome sequences of *A. albina* determined in this study and other 10 closely related species from GeneBank were used to perform phylogenetic analysis (Figure 1). The BI tree was constructed on CIPRES Portal using 13 PCGs, with the best-fit partitioning scheme and partition-specific models recommended by PartitionFinder (Lanfear et al. 2012). The phylogenetic analysis showed that *A. albina* was positioned near *A. remedios* within the genus of *Appias*, which indicated that our newly determined mitogenome sequence could meet the demands and explain some evolution issues.

**Figure 1.**
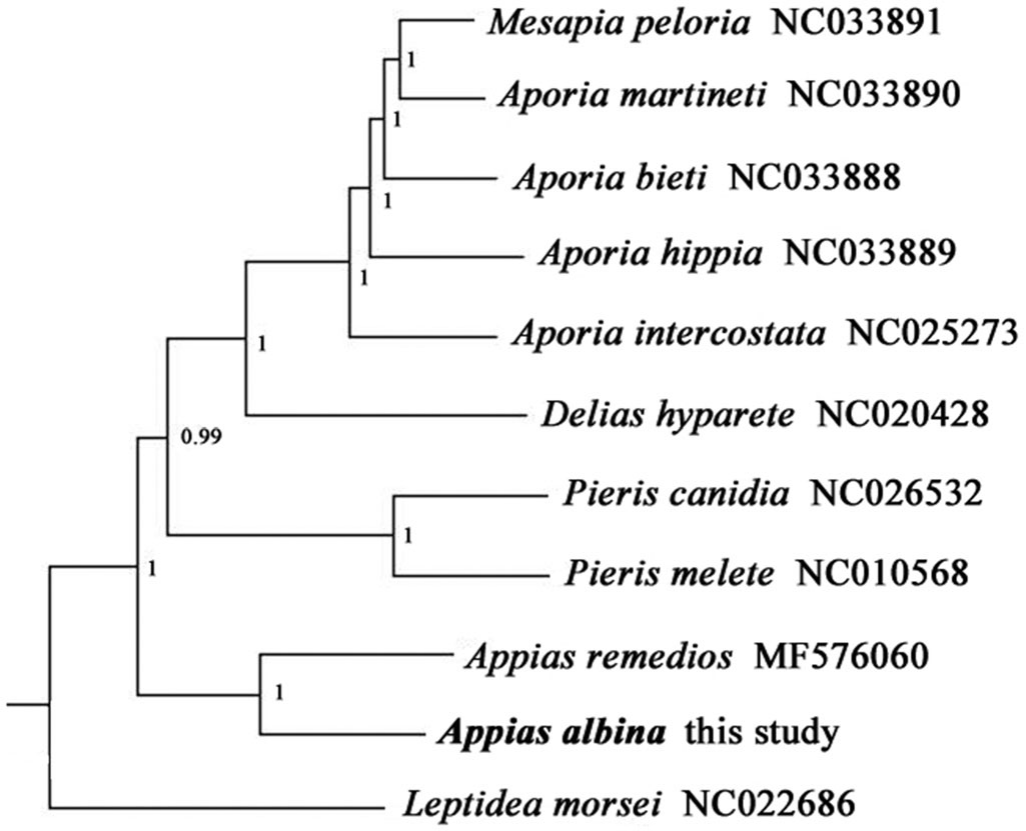
Phylogenetic tree obtained from BI analysis based on 13 concatenated mitochondrial PCGs. Numbers on node are posterior probability (PP).
